# Hypothalamic insulin resistance in type 2 diabetes is localized to the posterior hypothalamus

**DOI:** 10.1172/jci.insight.198707

**Published:** 2026-06-08

**Authors:** Hideyoshi Kaga, Akitoshi Ogawa, Takahiro Osada, Mai Kiya, Satoshi Oka, Yusuke Adachi, Mengping Yu, Shota Sakamoto, Saori Kakehi, Toshiki Kogai, Tsubasa Tajima, Hitoshi Naito, Naoaki Ito, Satoshi Kadowaki, Yuya Nishida, Ryuzo Kawamori, Seiki Konishi, Hirotaka Watada, Yoshifumi Tamura

**Affiliations:** 1 Department of Metabolism & Endocrinology,; 2 Department of Neurophysiology, and; 3 Sportology Center, Juntendo University Graduate School of Medicine, Tokyo, Japan.

**Keywords:** Endocrinology, Metabolism, Neuroscience, Diabetes, Insulin, Neuroimaging

## Abstract

Central insulin action in the brain is thought to contribute to metabolic regulation, but the specific hypothalamic nuclei affected in type 2 diabetes (T2D) remain poorly characterized. We performed high-resolution functional MRI (fMRI) during intranasal insulin administration to assess nucleus-level hypothalamic responses in 21 Japanese men with T2D and 20 individuals acting as healthy controls. In controls, insulin rapidly suppressed fMRI signals within 5 minutes in the posterior hypothalamic nucleus; this early suppression was not observed in T2D, indicating impaired hypothalamic insulin responsiveness. In an independent older cohort, structural MRI further revealed decreased gray matter volume in the corresponding posterior hypothalamus in participants with diabetes. These converging functional and structural findings implicate the posterior hypothalamus as a candidate locus associated with brain insulin resistance in T2D, warranting longitudinal and interventional validation.

## Abstract 2

In type 2 diabetes, hypothalamic insulin resistance is selectively localized to posterior and lateral hypothalamic nuclei, as revealed by high-resolution functional and structural brain imaging.

## Introduction

The incidence of type 2 diabetes (T2D) is increasing worldwide, making its prevention and treatment essential for preventing microvascular and macrovascular complications and mortality. The primary pathophysiology of T2D is insulin resistance and reduced insulin secretion. Although most studies have focused on peripheral insulin resistance in the liver, skeletal muscle, and adipose tissue, growing evidence indicates that hypothalamic insulin resistance plays a key role in contributing to the regulation of systemic energy balance, appetite, and glucose homeostasis. The hypothalamus plays a central integrative role in sensing hormonal and nutritional signals, including insulin, and in coordinating appropriate physiological responses (1, 2). Therefore, investigating hypothalamic insulin resistance is essential to fully understand the pathophysiology of T2D beyond its peripheral mechanisms.

Brain insulin sensitivity in humans has been assessed using functional MRI (fMRI) combined with intranasal insulin administration (3, 4), which bypasses the blood-brain barrier, enabling direct evaluation of central insulin responsiveness. Using this approach, studies have shown that obese individuals do not exhibit the typical decrease in hypothalamic activity following intranasal insulin administration. This impaired response is associated with peripheral insulin resistance and is indicative of “brain insulin resistance” (5). Brain insulin resistance has improved with interventions such as exercise (6), calorie restriction (7), and treatment with sodium glucose cotransporter 2 (SGLT2) inhibitors (8). These findings highlight the pathophysiological relevance and potential therapeutic modifiability of brain insulin resistance.

Despite these advances, most fMRI-based studies have treated the hypothalamus homogeneously or with limited subregions (7, 9). However, as each hypothalamic nucleus plays a distinct role in energy balance and metabolism, assessing insulin responsiveness at the nuclear level is crucial to capturing the spatial heterogeneity of hypothalamic function. Building on a previous imaging study that performed areal parcellation of the hypothalamus at a resolution of 1.25 mm and examined resting-state functional connectivity, we successfully identified 10 distinct hypothalamic foci using high-resolution fMRI (10–13). Importantly, these functional subregions have been shown to spatially correspond to the macroscopic anatomical locations of the hypothalamic nuclei. This nuclear-level functional mapping allows spatially specific evaluation of insulin effects in the human brain.

In the present study, we used high-resolution fMRI to examine the temporal and spatial patterns of hypothalamic responsiveness to intranasal insulin in persons with and without T2D (study 1). We identified specific hypothalamic subregions with altered insulin responses in patients with T2D and explored their association with clinical metabolic parameters. Furthermore, to evaluate whether these functional impairments are accompanied by structural changes, we analyzed the gray matter volume in a large cohort of older adults with and without diabetes (study 2). This two-pronged approach allowed us to assess both the functional and structural dimensions of brain insulin resistance and their clinical relevance. Collectively, our findings provide what we believe to be new insights into the nucleus-specific dynamics of hypothalamic insulin resistance and its potential anatomical correlates in diabetes.

## Results

### Study 1 — functional assessment of hypothalamic insulin response in middle-aged adults.

To investigate the differences in the insulin response in the brain between individuals with T2D and those without diabetes, we conducted a fMRI study (study 1) involving middle-aged participants. Table 1 lists the characteristics of study participants in the control and T2D groups. We initially enrolled 47 participants who underwent baseline anthropometric measurements (height, weight, BMI, and body fat percentage) and initiated the MRI protocol with intranasal insulin administration and blood sampling. Of these, 6 participants were excluded owing to inability to complete MRI scanning related to obesity or claustrophobia, severe fold-over artifacts, or excessive head motion during image acquisition. Consequently, a total of 41 participants were included in the final analyses, comprising 20 healthy individuals acting as controls and 21 persons with T2D. The mean duration of diabetes in the T2D group was 10.4 ± 7.7 years. Among the 21 individuals in the T2D group, 19 were on metformin, 12 on SGLT2 inhibitors, 6 on GLP-1 receptor agonists, 10 on DPP-4 inhibitors, 1 on sulfonylureas, 3 on glinides, 2 on α-glucosidase inhibitors, and 5 were receiving insulin therapy. The mean age of participants was 50 years. BMI, body fat percentage, fasting glucose levels, and fasting insulin levels were significantly higher in the T2D group (Table 1). HbA1c levels were 5.24% ± 0.25% in the control group and 6.88% ± 1.13% in the T2D group [t(20.843) = –6.32, *P* < 0.001]. Additionally, the T2D group exhibited significantly higher triglyceride and lower high-density lipoprotein cholesterol levels. Free fatty acid (FFA), high-molecular-weight adiponectin, C-reactive protein, and physical activity levels were similar between the groups. Leptin and glucagon levels were significantly elevated in patients with T2D. Regarding the surrogate markers of peripheral and adipose tissue insulin sensitivity, both the homeostasis model assessment of insulin resistance index and adipose tissue insulin resistance index were significantly higher in the T2D group, indicating reduced peripheral and adipose tissue insulin sensitivity than in the control group.

### Effects of intranasal insulin on plasma glucose, insulin, C-peptide, and FFA levels.

**Figure 1:**
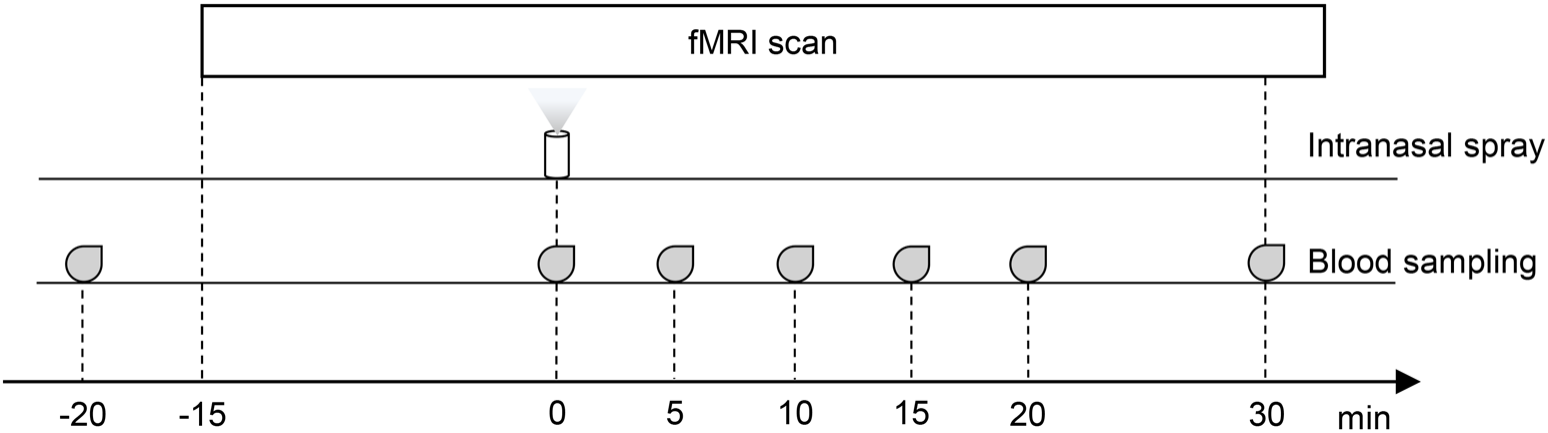
Timeline of the functional MRI protocol. Continuous BOLD fMRI scanning was performed from −15 to 30 minutes relative to intranasal insulin administration (as marked at 0 minutes). The first 15 minutes (−15 to 0 minutes) served as the baseline acquisition period, after which 160 IU of insulin was delivered intranasally while scanning continued without interruption. Peripheral blood samples for glucose, insulin, C-peptide, and free fatty acid assays were drawn at −20, 0, 5, 10, 15, 20, and 30 minutes and later aligned with the fMRI time series for statistical modeling.

**Figure 2:**
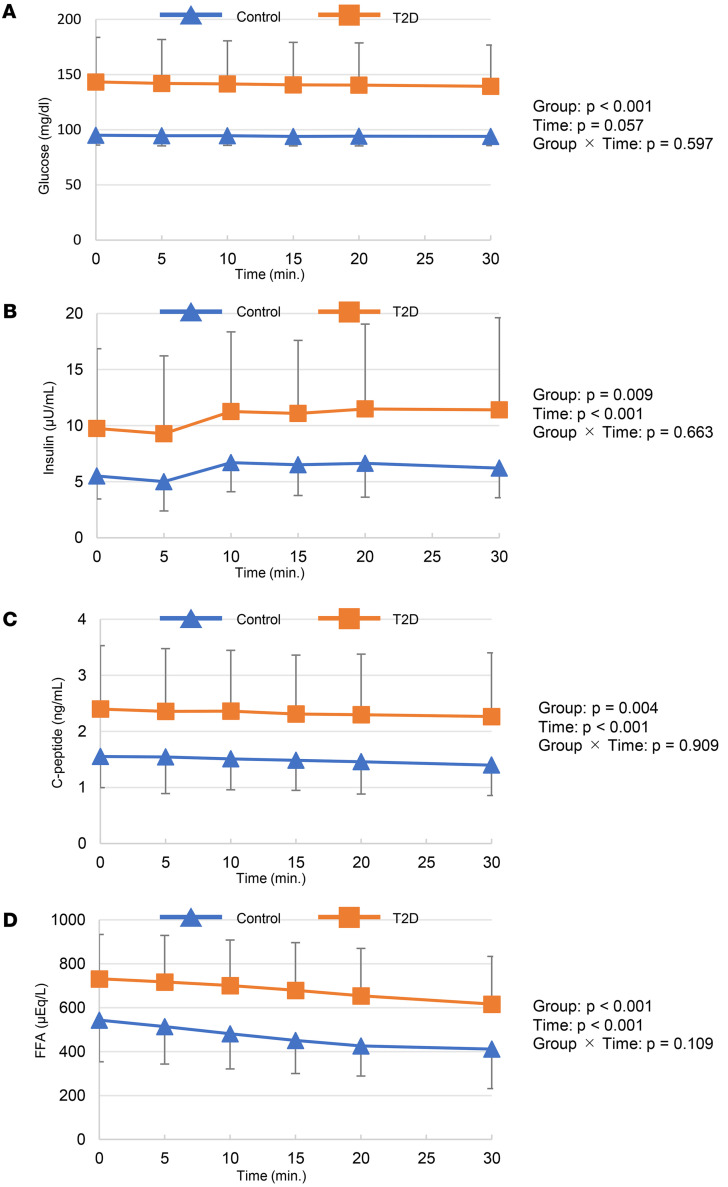
Peripheral metabolic responses to intranasal insulin. Plasma glucose (**A**), serum insulin (**B**), C-peptide (**C**), and free fatty acid (FFA) concentrations (**D**) measured before and after intranasal insulin administration in participants acting as controls and individuals with T2D. Data are expressed as mean ± SD for the control group (triangles) and the type 2 diabetes (T2D) group (rectangles). *P* values indicate the main effects of group and time and the group-by-time interaction derived from linear mixed-effects models.

We performed continuous blood oxygen level–dependent (BOLD) fMRI scanning from −15 to 30 minutes relative to intranasal insulin administration (Figure 1). Blood glucose, insulin, C-peptide, and FFA concentrations before and after insulin administration are shown in Figure 2, A–D. Time-course changes were analyzed using linear mixed-effects models with group, time, and their interaction as fixed effects and subject as a random effect. Significant main effects of group were observed for all 4 measures, with higher overall levels in individuals with T2D compared with participants acting as controls (all *P* < 0.01). A significant main effect of time was detected for serum insulin, C-peptide, and FFA concentrations (all *P* < 0.001), whereas the effect of time on plasma glucose did not reach statistical significance (*P* = 0.057). Specifically, serum insulin levels significantly increased following intranasal insulin administration, whereas C-peptide and FFA concentrations significantly decreased over time. No significant group-by-time interactions were observed for any of the metabolic variables (all *P* > 0.10). Collectively, these findings indicate that intranasal insulin induced modest time-dependent hormonal and lipid metabolic changes without significantly altering plasma glucose levels and that the magnitude and temporal patterns of these responses were comparable between the T2D and control groups during the first 30 minutes following administration.

### Effects of intranasal insulin on the MRI signal of the hypothalamic subregions.

**Figure 3:**
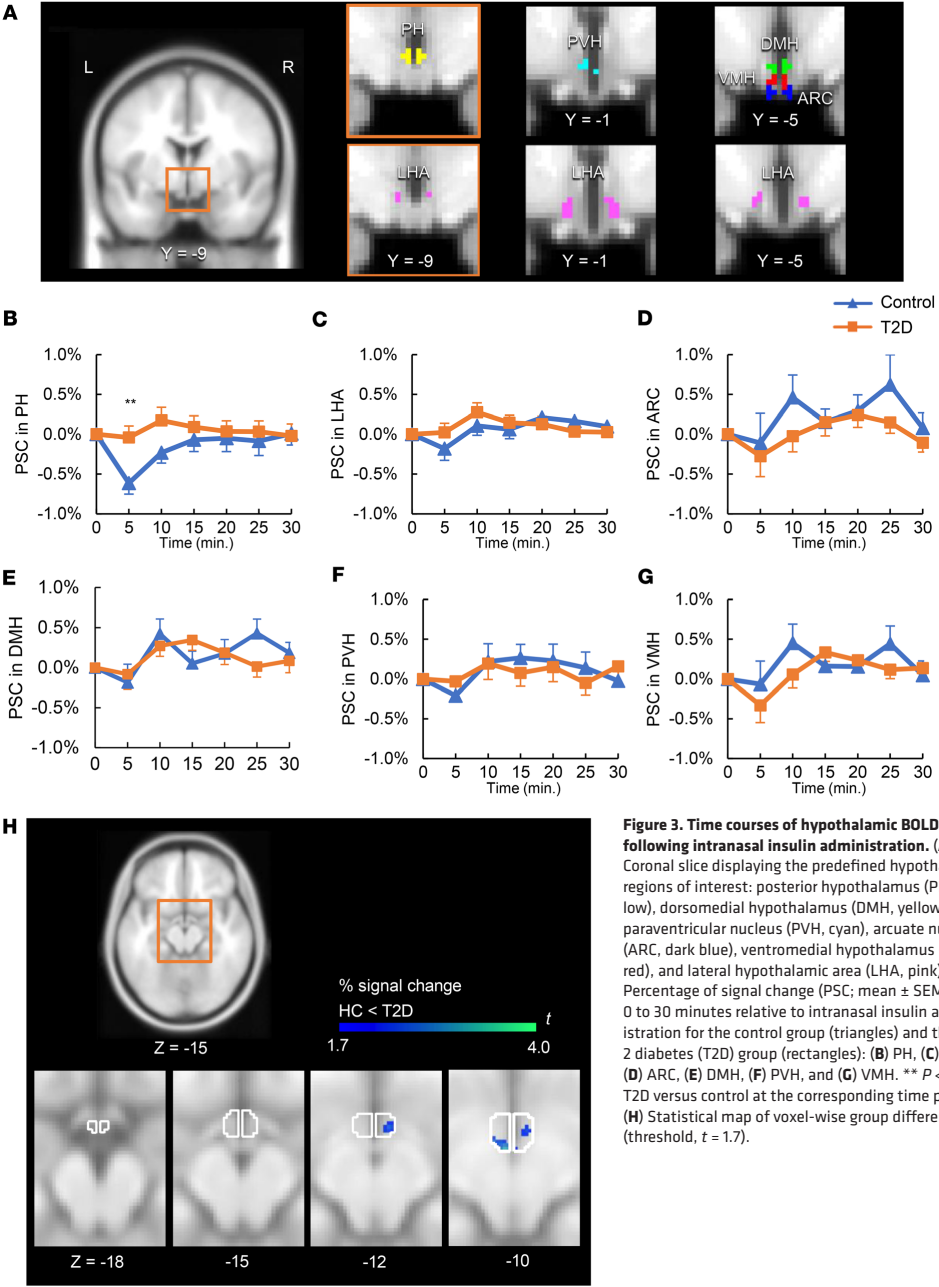
Time courses of hypothalamic BOLD signals following intranasal insulin administration. (**A**) Coronal slice displaying the predefined hypothalamic regions of interest: posterior hypothalamus (PH, yellow), dorsomedial hypothalamus (DMH, yellow-green), paraventricular nucleus (PVH, cyan), arcuate nucleus (ARC, dark blue), ventromedial hypothalamus (VMH, red), and lateral hypothalamic area (LHA, pink). (**B**–**G**) Percentage of signal change (PSC; mean ± SEM) from 0 to 30 minutes relative to intranasal insulin administration for the control group (triangles) and the type 2 diabetes (T2D) group (rectangles): (**B**) PH, (**C**) LHA, (**D**) ARC, (**E**) DMH, (**F**) PVH, and (**G**) VMH. ** *P* < 0.01 T2D versus control at the corresponding time point. (**H**) Statistical map of voxel-wise group difference (threshold, *t* = 1.7).

MRI signal changes after nasal insulin administration are shown in Figure 3. Our primary analysis focused on functional hypothalamic subregions implicated in metabolic regulation. These subregions were selected from subdivisions defined a priori based on our previous resting-state functional connectivity parcellation (10–13). For the present study, we examined 5 medial subregions and 1 lateral region: the posterior hypothalamic nucleus (PH), the arcuate nucleus of the hypothalamus (ARC), the dorsomedial nucleus of the hypothalamus (DMH), the paraventricular nucleus of the hypothalamus (PVH), the ventromedial nucleus of the hypothalamus (VMH), and the lateral hypothalamic area (LHA) (Figure 3A). Among these regions, rapid signal suppression was observed only in the PH in the control group, whereas this response was not observed in the T2D group (Figure 3B). This group difference in the PH signal change at 5 minutes was statistically significant (t = 2.86, *P* = 0.0067, unpaired *t* test) and survived Bonferroni correction for 6 comparisons (corrected significance threshold, *P* < 0.0083 [0.05/6]). This finding highlights the region- and time-specific nature of central insulin responsiveness and suggests that the early-phase response is impaired in T2D. In contrast, no significant group differences were observed in the remaining 5 regions (LHA, ARC, DMH, PVH, and VMH) during either the early phase or the entire 30-minute observation period (Figure 3, C–G). Consistent with the region of interest–based (ROI-based) findings, a voxel-wise comparison at 5 minutes demonstrated that the group difference was primarily localized to the posterior hypothalamus (Figure 3H). Because the LHA is relatively large, we additionally conducted an exploratory analysis subdividing the LHA into anterior, tuberal, and posterior portions (LHAa, LHAt, and LHAp) (Supplemental Figure 1A; supplemental material available online with this article; https://doi.org/10.1172/jci.insight.198707DS1). In this subregional analysis, modest signal suppression was observed in the control group within the LHAp at 5 and 10 minutes, whereas no such response was observed in the T2D group (Supplemental Figure 1, B–D).

To confirm the robustness of this primary PH finding, we performed two sensitivity analyses focusing on the 5-minute PH response. First, to minimize potential spatial distortion due to spatial normalization, we inversely transformed the Montreal Neurological Institute–defined (MNI-defined) PH ROI into each participant’s native space and reextracted the fMRI signals. This native-space analysis yielded consistent results, showing significantly greater signal suppression in participants acting as controls than in those with T2D (Supplemental Figure 2A). Second, to ensure independence from the functional connectivity-based parcellation scheme, we repeated the analysis using an anatomically defined PH mask derived from a high-resolution structural atlas (14). This analysis likewise confirmed the significant group difference (Supplemental Figure 2B). Together, these analyses demonstrate that the observed PH functional impairment in T2D is robust and not attributable to spatial normalization artifacts or ROI definition.

To verify that the early PH signal suppression was insulin-specific rather than a nonspecific effect of nasal administration, we evaluated the PH signal in an independent healthy cohort (*n* = 7) that received intranasal distilled water. Distilled water did not induce comparable PH signal suppression. A direct comparison at 5 minutes showed significantly greater PH suppression in the insulin-treated control group than in the water-treated cohort (t = 2.10, *P* = 0.046, unpaired *t* test) (Supplemental Figure 3).

Finally, within the T2D group, we performed stratified analyses comparing participants with T2D who were taking SGLT2 inhibitors (*n* = 12) and those with T2D who were not receiving these agents. No significant differences in hypothalamic MRI signal changes were observed between these subgroups, and no PH signal suppression was detected in either subgroup (t = –0.974, *P* = 0.342, unpaired *t* test), suggesting that SGLT2 inhibitor use did not drive the observed group differences.

### Association between hypothalamic signal changes and metabolic parameters.

To further investigate the relationship between insulin-induced hypothalamic signal changes and metabolic characteristics, multiple regression analyses were performed focusing on the primary functional finding (i.e., PH signal change at 5 minutes). As shown in Table 2, no statistically significant associations were observed between PH signal changes at 5 minutes and any metabolic parameters after adjustment for glycemic status (T2D vs. control). Testing of group-by-metabolic parameter interaction terms in full models also yielded no significant interactions. Additionally, exploratory analyses of the LHAp responses are presented in Supplemental Table 1. LHAp signal changes at 5 minutes were positively associated with fasting plasma glucose and HbA1c levels. Similarly, LHAp signal changes at 10 minutes were positively associated with fasting plasma glucose.

### Study 2 — gray matter volume in the posterior hypothalamus is decreased in individuals with diabetes.

To assess whether the structural alterations in the hypothalamus were accompanied by the functional differences observed in study 1, we analyzed brain MRI data from another cohort of older adults. The second cohort (study 2) (15) comprised persons with and without diabetes, allowing us to examine differences in hypothalamic gray matter volume. The total number of participants in this cohort was 1,629. Of these, 1,609 individuals with available gray matter volume data were included in this analysis. Among them, 209 were classified into the diabetes group and 1,400 into the nondiabetes group. The baseline characteristics of these participants are presented in Supplemental Table 2. In addition, detailed information on glucose-lowering medications used by participants with diabetes in the study 2 cohort is provided in Supplemental Table 3. Compared with the nondiabetes group, the diabetes group was significantly older, had a lower proportion of women, higher BMI and body fat percentage, and a lower skeletal muscle mass index. The prevalence of hypertension, dyslipidemia, and ischemic heart disease was also higher in the diabetes group. However, there were no significant differences in the prevalence of cerebrovascular disease, cognitive function, physical activity, or dietary intake. The mean ± SD fasting plasma glucose and HbA1c levels in the diabetes group were 130.6 ± 23.3 mg/dL and 6.9% ± 0.7%, respectively.

**Figure 4:**
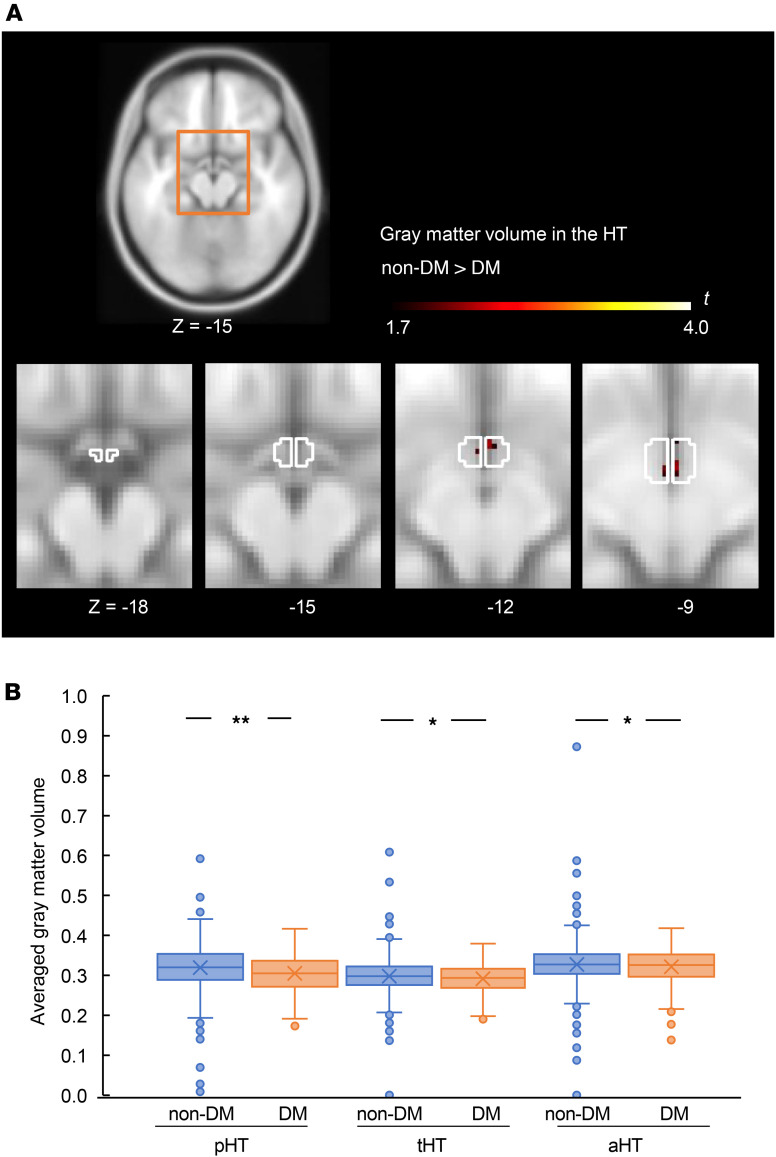
Hypothalamic gray matter volume in the Bunkyo Health Study cohort (study 2). (**A**) Axial T1-weighted slice showing the hypothalamus and its 3 anatomical subdivisions: anterior, tuberal, and posterior. (**B**) Box plots comparing gray matter volume in each subdivision between the nondiabetes group (non-DM, blue) and the diabetes group (DM, orange). Each box represents the interquartile range (IQR), the horizontal lines within boxes indicate the median, and the whiskers extend to the minimum and maximum values within 1.5×IQR. Data points beyond the whiskers are plotted individually as outliers. The cross marks (×) represent the mean. Group differences were tested with a *t* test (DM vs. non-DM). *P < 0.05, **P < 0.01.

While nucleus-level subregion analysis was emphasized in study 1, the 0.3 T MRI used in study 2 did not permit that level of resolution. Therefore, for regional analysis, the hypothalamus was divided into 3 anatomically defined regions: the anterior (aHT), tuberal (tHT), and posterior (pHT) hypothalamus. Notably, the pHT corresponds anatomically to the PH and LHAp regions evaluated in study 1. An analysis of covariance adjusted for age and sex revealed that gray matter volume in the hypothalamus was significantly lower in the diabetes group compared with that in the nondiabetes group across all 3 regions (Figure 4, A and B). Among these, the reduction in pHT was more pronounced in the diabetes group [F(1,1604) = 6.788, *P* = 0.009], compared with tHT (F = 5.269, *P* = 0.022) and aHT (F = 4.378, *P* = 0.037) groups, indicating that the posterior hypothalamus may be more sensitive to diabetes-related changes in gray matter volume (Figure 4B), corresponding to the functional deficits identified in study 1. In a supplementary sex-stratified analysis, a significant reduction in pHT volume was observed in male participants (F = 4.752, *P* = 0.030), whereas no statistically significant differences were detected in female participants (F = 2.177, *P* = 0.140). However, the number of women with diabetes was relatively small, limiting statistical power to detect sex-specific effects. Furthermore, to determine whether the functional impairment observed in study 1 was accompanied by macroscopic structural atrophy, we additionally performed volumetric analyses in the study 1 cohort using the same macroscopic segmentation scheme. As shown in Supplemental Figure 4A, no statistically significant differences in the gray matter volume of the pHT (or other regions) were observed between the T2D and control groups in this middle-aged cohort. Notably, when applying 3-region segmentation to the fMRI data from study 1, only the posterior hypothalamus showed a significant difference in signal change between the T2D and control groups (Supplemental Figure 4B), suggesting a functional correlation with the structural vulnerability observed in study 2.

## Discussion

Although hypothalamic insulin resistance has long been associated with metabolic disorders, such as T2D (2), the specific nuclei responsible have not been identified. In this study, we investigated changes in hypothalamic brain activity following intranasal insulin administration in individuals with and without T2D, aiming to clarify insulin resistance at the level of distinct hypothalamic subregions. Our results revealed that in participants without diabetes, MRI signals in the PH were rapidly suppressed, whereas no significant change in the signal was observed in the T2D group. In addition, a separate structural MRI analysis of an independent cohort of older adults demonstrated that individuals with diabetes exhibited significantly reduced gray matter volume in the corresponding posterior hypothalamus. Taken together, these findings highlight the posterior hypothalamus as a critical locus for the insulin response and hypothalamic insulin resistance.

The PH is considered essential for thermoregulation, sympathetic outflow, and energy expenditure (16). Our primary analysis newly identified the PH as the specific locus exhibiting early functional impairment in insulin responsiveness in individuals with T2D. While the involvement of the PH in T2D has previously received less attention compared with other classical metabolic nuclei, our findings suggest that it plays a pivotal role in central insulin action. Further investigation is required to elucidate the precise biological mechanisms and pathophysiological importance of insulin’s effect on the posterior hypothalamus. In addition to the primary finding in the PH, our exploratory subregional analysis revealed that MRI signals in the LHAp were also rapidly modulated following intranasal insulin administration in participants in the control group, a response that was absent in the T2D group. This underscores the critical role of the LHA in glucose metabolism and energy homeostasis (17). Recent single-cell sequencing studies have identified numerous transcriptionally distinct neuronal populations within the LHA (17), some of which exert opposing effects on feeding, energy expenditure, and long-term energy balance. For instance, certain glutamatergic neurons in the LHA suppress food intake (18, 19), whereas GABAergic neurons promote feeding behavior (20). Therefore, based on the current fMRI results alone, no definitive conclusion can be drawn regarding whether insulin-induced suppression of MRI signals increases or decreases food intake and energy balance. Notably, the rapid onset of these insulin responses contrasts with previous MRI studies that assessed later, discrete time points (e.g., 30 minutes) (3, 4). Our continuous scanning design effectively captured these early dynamics. Biologically, this rapid central action is plausible, as intranasal delivery provides fast access via olfactory and trigeminal pathways, although precise human transport kinetics remain unverified.

An important consideration from our exploratory analyses is the positive correlation between the LHAp signal changes and fasting plasma glucose level and HbA1c levels. The LHA contains diverse populations of glucose-sensing neurons, including both glucose-excited and glucose-inhibited neurons, as well as neurons expressing insulin receptors (21, 22). This cellular diversity positions the LHA as a central integrative hub for metabolic and neuroendocrine regulation. In humans, blood glucose levels have been reported to influence the blood-to-brain transfer of insulin (23), suggesting that glucose availability may function as a gatekeeper for central insulin action. Therefore, the blunted LHAp response observed in T2D may disrupt this regulatory system associated with elevated fasting plasma glucose levels.

In contrast to the PH and LHAp, we observed no significant early-phase group differences in MRI signal changes in the ARC or the PVH. Although these nuclei are central to metabolic regulation (1), evidence regarding insulin’s direct acute effects on these regions, particularly in humans, is limited. To date, no human neuroimaging studies have specifically demonstrated insulin-induced responses in either nucleus. In rodents, the role of insulin in these regions involves complex, and sometimes opposing, actions on AgRP/NPY and POMC neurons (24–27). Furthermore, these circuits integrate multiple peripheral signals, including nutrients and hormones, which means their dynamic responses are highly complex. It is also important to acknowledge that MRI signals do not capture all aspects of neuronal activity (28). For instance, the unique anatomical environment of the ARC, which is surrounded by cerebrospinal fluid may attenuate MRI signals. In addition, brain areas dominated by inhibitory neurons, such as GABAergic populations, may not exhibit a straightforward relationship between neuronal firing and BOLD signal changes. Therefore, the absence of early signal differences in hypothalamic subregions such as the ARC or PVH does not diminish their importance in glucose and feeding regulation; rather, it suggests a unique responsiveness of the PH and LHAp to intranasal insulin administration observed in this study.

In addition to functional alterations, our study provides what we believe to be novel insights into the timeline of structural changes associated with brain insulin resistance. In our middle-aged cohort, (study 1), volumetric analysis revealed no significant macroscopic structural differences in the posterior hypothalamus between the T2D and control groups. However, our structural MRI of a much larger and older population (study 2) demonstrated that older adults with diabetes exhibited a pronounced reduction in gray matter volume specifically in the corresponding posterior hypothalamus. Rather than contradicting our functional findings, we believe these combined results provide pathological insights. They suggest that functional impairments in hypothalamic insulin responsiveness precede measurable macroscopic structural atrophy, which likely becomes evident only later in life after a prolonged metabolic burden. Moreover, our previous research demonstrated that even middle-aged individuals with metabolic syndrome exhibit microstructural changes in hypothalamic white matter (29), and obesity is associated with increased gliosis and neuroinflammation in the hypothalamus (30). Together, these findings suggest that altered functional insulin responsiveness in the posterior hypothalamus may represent an early neuropathological feature of diabetes, eventually leading to macroscopic remodeling.

This study has several limitations. First, a within-subject placebo control condition (e.g., using a crossover design) was not implemented in the main study cohort. To address potential nonspecific effects related to nasal administration (e.g., sensory stimulation, mechanical effects of spraying, or expectancy), we conducted a control experiment using intranasal distilled water in an independent healthy individual acting as a control (*n* = 7). While this separate cohort did not exhibit the early PH signal suppression observed in the insulin-treated group, this between-subjects comparison has inherent limitations. Future studies employing a randomized, double-blind, within-subject crossover design are necessary to definitively isolate the pharmacological effects of central insulin from procedural artifacts. Second, while our primary analysis of the 6 predefined functional subregions was rigorously controlled for multiple comparisons, our subregional analysis of the LHA and its association with fasting glucose was exploratory. Therefore, these exploratory findings should be interpreted cautiously and require independent replication in future adequately powered studies. Third, the heterogeneity of diabetes medications in the T2D group must be considered in study 1. Although our stratified analysis suggested that the presence or absence of SGLT2 inhibitors did not drive the observed group differences in PH signal suppression, the limited sample size prevented a comprehensive evaluation of all medication classes or insulin therapy. Furthermore, diabetes duration was not systematically collected for study 2. Because this retrospective dataset was originally designed for population-level morphometric analyses, we could not evaluate how the temporal progression of diabetes influences macroscopic structural remodeling in the hypothalamus. Fourth, study 1 included only male participants. Therefore, the generalizability of our findings to females remains unknown. Future studies are warranted to investigate potential sex differences in central insulin responsiveness. Fifth, caffeine and nicotine intake prior to the study day was not systematically assessed. As both factors may influence hypothalamic activity and neurovascular responses, their potential effects on the observed MRI signals cannot be fully excluded. Sixth, while our study focused solely on the hypothalamus, it is increasingly recognized that hypothalamic neurons project to other brain regions, forming complex neural circuits. Further investigation of these broader neural networks is warranted to fully understand the central regulation of metabolism. Finally, owing to the cross-sectional nature of our study, establishing causal relationships was challenging. Future research should explore whether diabetes medications and lifestyle interventions, such as diet and exercise therapy, influence neuronal activity in the posterior hypothalamus, as observed in the present study.

In conclusion, our high-resolution fMRI study revealed a selective, early-phase blunting of central insulin responsiveness in individuals with T2D, primarily localized to the PH. Importantly, this functional impairment corresponded anatomically to macroscopic structural atrophy observed in the posterior hypothalamus of an older adult cohort with diabetes. These converging findings provide what we believe to be novel insights into the development and progression of T2D. Further investigations into the precise neurocircuitry of these specific nuclei and their potential as therapeutic targets for metabolic disorders are highly warranted.

## Methods

### Sex as a biological variable

In study 1, only male participants were included to minimize variability related to sex-specific hormonal differences; therefore, the generalizability of these findings to females remains unknown. In study 2, both male and female participants were included. Sex was incorporated as a covariate in the statistical analyses, and additional sex-stratified analyses were performed to explore potential sex-specific differences. However, the number of female participants with diabetes was relatively small, which may have limited the statistical power to detect sex-specific effects.

### Study 1

#### Study participants.

We recruited 24 Japanese men with T2D and 23 Japanese men without diabetes. All participants were right-handed and aged between 40 and 64 years. Participants with acute cardiovascular disease, chronic lung disease, cancer, renal failure, serious hepatic dysfunction, hyperthyroidism, dementia, or mental illnesses were excluded.

A formal a priori power calculation was not performed, and a single primary endpoint was not prespecified. As this study was designed as an exploratory investigation to characterize hypothalamic responses to intranasal insulin using high-resolution fMRI, the sample size was determined pragmatically based on methodological standards in human neuroimaging research. We targeted 20 participants per group, consistent with recommendations suggesting that a minimum sample size of approximately *n* = 20 is required to detect biologically meaningful effects (31). This target is also in line with the typical sample sizes reported for neuroimaging studies (32). To obtain 20 analyzable datasets per group, we prospectively recruited 47 participants, accounting for an anticipated exclusion rate of approximately 15%.

Of the 47 initially enrolled participants, 6 were excluded prior to group-level analyses due to an inability to complete MRI scanning (e.g., related to obesity or claustrophobia), severe fold-over artifacts, or excessive head motion during image acquisition. Consequently, 41 participants were included in the final analyses, comprising 21 individuals with T2D and 20 nonobese (BMI < 25 kg/m^2^) healthy individuals acting as controls without diabetes.

#### Study design.

After the screening session, participants visited our institution on a separate day for the experimental session. Participants were instructed to fast from 9:00 PM on the evening prior to the experiment, and all fMRI sessions were conducted starting at approximately 7:00 AM, ensuring a fasting duration of at least 10 hours. During the fasting period, only water intake was permitted. Participants with diabetes continued their usual glucose-lowering medications during the study period. However, on the day of the fMRI session, all medications were withheld in the morning to avoid acute pharmacological effects during scanning. No extended washout period was implemented.

Following an overnight fast, fMRI was performed in combination with intranasal insulin administration. The study protocol is illustrated in Figure 1. An intravenous cannula was inserted into the forearm vein for blood sampling. Participants received an intranasal insulin spray at time 0 (15 minutes after the start of the MRI scan). One puff of the 0.1 mL spray solution contained 10 IU human insulin (100 IU/mL Humulin R; Eli Lilly and Company). Eight puffs were administered to each nostril for approximately 2.5 minutes, resulting in a total dose of 160 IU insulin (33). Blood samples were obtained at –20, 0, 5, 10, 15, 20, and 30 minutes. Following the fMRI scan, total body fat content was measured using the bioimpedance method (InBody; BIOSPACE). Dietary habits and physical activities were assessed using the Brief Diet History Questionnaire (34, 35) and the International Physical Activity Questionnaire, respectively (36, 37).

#### MRI acquisition and data analysis.

All MRI data were acquired using a 3-T MRI scanner (Siemens Prisma) with a 32 ch head coil at Juntendo University Hospital. T1-weighted structural images were obtained using a 3D magnetization-prepared rapid gradient echo (MPRAGE) sequence (resolution, 0.8 × 0.8 × 0.8 mm^3^). Functional images were obtained using a multiband gradient-echo (GRE) echo-planar imaging (EPI) sequence (repetition time, 2.5 s, echo time, 20 ms; flip angle, 77º, in-plane field of view [FOV], 180 mm × 180 mm; matrix size, 144 × 144; 108 contiguous slices with no gap; phase encoding direction, posterior-anterior; no parallel acquisition; and multiband factor, 3) (38, 39). In total, 1,140 volumes were acquired from each participant. In addition, single images with anterior-posterior and posterior-anterior encoding directions were acquired using a spin-echo EPI sequence with the same FOV and resolution as the GRE EPI sequence. These images were used to perform top-up distortion correction (40). At the beginning of each fMRI session, a single-band GRE EPI image with the same dimension and resolution as the multiband GRE EPI was acquired as a reference for motion correction and coregistration with structural images (41).

Preprocessing was performed using FSL (https://fsl.fmrib.ox.ac.uk/fsl/fslwiki). Functional images were realigned, distortion-corrected, and then coregistered to each participant’s high-resolution T1-weighted structural image. Structural images were nonlinearly normalized to the MNI standard space, and the resulting transformation parameters were applied to the functional data. Quality control was conducted systematically for all participants through visual inspection of the alignment between normalized functional images and the MNI template, with specific attention to anatomical landmarks surrounding the hypothalamus.

Head motion was quantified using frame-wise displacement (FD) (42). Participants were excluded if motion resulted in severe visible artifacts or gross misalignment, leading to the exclusion of 2 participants prior to group-level analyses. The mean (± SEM) FD was 0.17 (± 0.02) mm in the control group and 0.21 (± 0.02) mm in the T2D group, with no significant between-group difference (t = 1.89, *P* = 0.07). No volume-level censoring (scrubbing) was applied, as we aimed to preserve the continuous temporal structure of the data for assessing the gradual pharmacological effects of intranasal insulin. Instead, to compute fMRI signal changes, the 6 head motion parameters obtained during realignment were regressed out from the fMRI signals at the individual level to mitigate motion-related artifacts. Cardiac and respiratory signals were not recorded during acquisition; therefore, physiological noise regressors were not included.

The average of the cleaned fMRI signal from –15 minutes to 0 minutes relative to insulin administration was used as the baseline in each participant. Signal changes compared with this baseline were calculated every 5 minutes following intranasal insulin spray. For example, the signal change at 5 minutes was the average of the fMRI signal from 2.5 minutes to 7.5 minutes divided by the baseline. Similarly, the signal changes at 10, 15, 20, 25, and 30 minutes were calculated.

The ROIs were defined a priori based on our previous resting-state fMRI parcellation studies (10–13) using a boundary mapping technique (43–50). The ROI definitions were established independently of the present dataset. The primary analysis focused on hypothalamic subregions involved in metabolic regulation, including 5 medial subregions and 1 lateral region: the PH, the ARC, the DMH, the PVH, and the VMH as well as the LHA. The number of voxels was as follows: PH (L: 5, R: 5), ARC (L: 6, R: 5), DMH (L: 5, R: 5), PVH (L: 5, R: 5), VMH (L: 6, R: 5), and LHA (L: 43, R: 45). ROI delineation was completed prior to any group-level statistical analysis, and the investigator responsible for ROI preparation was blinded to participant group assignment.

#### Biochemical tests.

Plasma insulin and leptin concentrations were determined using radioimmunoassay (LINCO Research). Plasma glucose and C-peptide concentrations were evaluated by ultraviolet and electrochemiluminescence immunoassays, respectively. Serum lipid levels, including FFA, high- and low-density lipoprotein cholesterol, and triglycerides, were measured using enzymatic methods (SRL Inc.). Serum adiponectin concentrations were measured using a 30-enzyme-linked immunosorbent assay (Daiichi Pure Chemicals). The homeostasis model assessment of insulin resistance was performed as previously described (51). We also evaluated the degree of insulin resistance in the adipose tissue using the adipose tissue insulin resistance index (fasting insulin levels × fasting FFA) (52).

#### Supplementary control experiment.

To control for potential nonspecific effects of the intranasal spray procedure, such as sensory stimulation, or expectancy, we conducted an additional fMRI experiment in an independent cohort of 7 healthy volunteers (mean age 32.0 ± 4.8 years; all male, with a mean BMI of 21.5 ± 2.1 kg/m²). These participants underwent an identical fasting and fMRI scanning protocol as described above, with two exceptions: they received an intranasal spray of distilled water (1.6 mL total volume) instead of insulin, and peripheral blood sampling was not performed. Data acquisition, preprocessing, and extraction of the percentage of signal change in the hypothalamic ROI were conducted using the same procedures as in the main experiment.

### Study 2

#### Study participants.

To complement the functional data with structural insights, we analyzed a separate community-based cohort (study 2) using brain MRI to compare hypothalamic gray matter volume between individuals with and without diabetes. Baseline data were obtained from the Bunkyo Health Study, a prospective cohort of 1,629 participants aged 65–84 years living in Bunkyo-ku, an urban area in Tokyo, Japan (15, 53). All participants underwent a 75-g oral glucose tolerance test (OGTT). Diabetes was defined as a combination of fasting plasma glucose ≥126 mg/dL and/or a 2-h glucose level ≥200 mg/dL after the 75-g OGTT, and HbA1c ≥6.5% or current treatment with medication for diabetes mellitus (54). Participants were classified into diabetes or nondiabetes groups accordingly. We evaluated the volume of the gray matter in the hypothalamus using structural brain MRI.

#### MRI procedures.

We evaluated structural head images, including the entire brain, using a 0.3-T MRI scanner (AIRIS Vento, Hitachi). T1-weighted images were obtained utilizing a 3D GRE sequence with inversion recovery (repetition time [TR], 25 ms; echo time [TE], 5.8 ms; inversion time [TI], 600 ms; flip angle [FA],12°; FOV, 200 × 250 × 250 mm³; resolution, 0.98 × 0.98 × 2.0 mm³; slice orientation, sagittal). Previous research has confirmed the reliability of gray matter volume analysis using images obtained from the 0.3-T MRI scanner, even when compared with those obtained using a 3-T scanner (55).

### Statistical analysis

Data are presented as mean ± SD or mean ± SEM, as appropriate. Log-transformed values were used to approximate normal distributions. No data points were excluded based on statistical outlier criteria; all metabolic and imaging data were retained, provided they met the predefined quality control procedures.

In study 1, differences in baseline characteristics between the two groups (control and T2D) were evaluated using the unpaired Student’s *t* test or Mann-Whitney *U* test, as appropriate. All *t* tests were 2-tailed. Time-course data for plasma glucose, serum insulin, C-peptide, and FFA were analyzed using linear mixed-effects models. Group (control and T2D), time (0, 5, 10, 15, 20, and 30 minutes), and their interaction were included as fixed effects, while subject was treated as a random effect to account for within-participant repeated measurements. An unstructured covariance matrix was applied to model the correlations among repeated measures over time. Estimated marginal means were calculated, and post hoc comparisons were performed with Bonferroni’s correction when appropriate. To examine associations between hypothalamic signal changes and metabolic parameters, we utilized multiple regression models adjusting for group status (T2D vs. control), focusing on the 5-minute PH response. In study 2, the differences in baseline characteristics between the persons with and without diabetes groups were assessed using unpaired *t* tests for continuous variables and χ^2^ tests for categorical variables. All *t* tests were 2-tailed. For comparisons of gray matter volume in the hypothalamic subregions, an analysis of covariance (1-way ANCOVA) was performed with adjustments for age and sex. A *P* value of less than 0.05 was considered statistically significant. All statistical tests were 2-tailed. SPSS Statistics for Windows, version 29.0. (IBM Corp.) was used for analysis.

### Study approval

All studies involving human participants were reviewed and approved by the Ethics Committee of Juntendo University. For study 1, the protocol was approved under approval number M20-0076. All participants provided written informed consent prior to participation in the study. For study 2, the protocol was approved in September 2015 (approval no. 2015061; latest revised version no. M15-0057-M09). All participants provided written informed consent and were informed of their right to withdraw from the study at any time. All studies were conducted in accordance with the principles of the Declaration of Helsinki.

### Data availability

The data supporting the findings of this study are available within the manuscript and its Supporting Data Values file. Additional data are not publicly available due to restrictions related to human subject privacy but are available from the corresponding author upon reasonable request, in accordance with institutional guidelines.

## Author contributions

HK, MK, AO, TO, and YT performed the research and contributed to the study design, data collection, interpretation of the results, and writing and editing of the manuscript. SO, YA, MY, SS, S Kakehi, TK, TT, HN, NI, and S Kadowaki participated in the data collection and analysis and contributed to the discussion. YN, RK, and S Konishi contributed to the discussion. HW contributed to the study design and reviewed and edited the manuscript.

## Conflict of interest

The authors have declared that no conflict of interest exists.

## Funding support

- Ministry of Education, Culture, Sports, Science, and Technology of Japan grants (KAKENHI: JP21H03380 to YT; JP22K07334 to AO; JP21K07255 to TO; JP23K27474 to S Konishi).
- Japan Health Promotion Foundation (to HW).
- Japan Diabetes Foundation (to HK).
- AMED-CREST (JP24gm2010005 to AO and S Konishi).
- Watanabe Foundation (to TO).

## Supplementary Material

Supplemental data

Supporting data values

## Figures and Tables

**Table 1:**
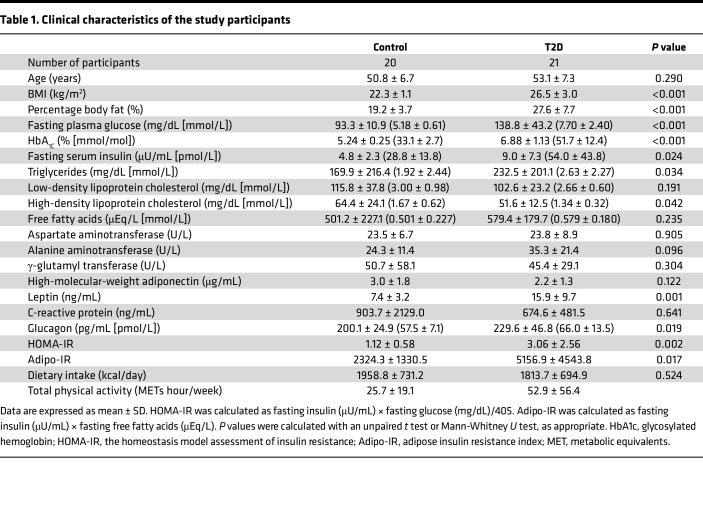
Clinical characteristics of the study participants

**Table 2:**
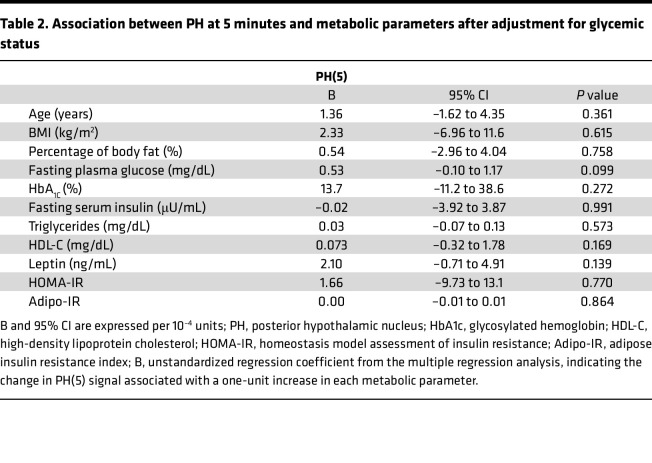
Association between PH at 5 minutes and metabolic parameters after adjustment for glycemic status

## Version 1 06/08/2026

Electronic publication

## References

[1] Chen W (2022). Insulin action in the brain: cell types, circuits, and diseases. Trends Neurosci.

[2] Heni M (2024). The insulin resistant brain: impact on whole-body metabolism and body fat distribution. Diabetologia.

[3] Nijssen KMR (2023). Effects of intranasal insulin administration on cerebral blood flow and cognitive performance in adults: a systematic review of randomized, placebo-controlled intervention studies. Neuroendocrinology.

[4] Heni M (2017). Hypothalamic and striatal insulin action suppresses endogenous glucose production and may stimulate glucose uptake during hyperinsulinemia in lean but not in overweight men. Diabetes.

[5] Kullmann S (2015). Selective insulin resistance in homeostatic and cognitive control brain areas in overweight and obese adults. Diabetes Care.

[6] Kullmann S (2022). Exercise restores brain insulin sensitivity in sedentary adults who are overweight and obese. JCI Insight.

[7] Teeuwisse WM (2012). Short-term caloric restriction normalizes hypothalamic neuronal responsiveness to glucose ingestion in patients with type 2 diabetes. Diabetes.

[8] Kullmann S (2022). Empagliflozin improves insulin sensitivity of the hypothalamus in humans with prediabetes: a randomized, double-blind, placebo-controlled, phase 2 trial. Diabetes Care.

[9] Lundqvist MH (2019). Is the brain a key player in glucose regulation and development of type 2 diabetes?. Front Physiol.

[10] Osada T (2017). Functional subdivisions of the hypothalamus using areal parcellation and their signal changes related to glucose metabolism. Neuroimage.

[11] Ogawa A (2020). Connectivity-based localization of human hypothalamic nuclei in functional images of standard voxel size. Neuroimage.

[12] Ogawa A (2022). Hypothalamic interaction with reward-related regions during subjective evaluation of foods. Neuroimage.

[13] Rison NO (2024). Stereotaxic coordinates of human hypothalamic nuclei used for region of interest analyses in functional magnetic resonance imaging. Juntendo Med J.

[14] Neudorfer C (2020). A high-resolution in vivo magnetic resonance imaging atlas of the human hypothalamic region. Sci Data.

[15] Someya Y (2019). Skeletal muscle function and need for long-term care of urban elderly people in Japan (the Bunkyo Health Study): a prospective cohort study. BMJ Open.

[16] Cavdar S (2001). The afferent connections of the posterior hypothalamic nucleus in the rat using horseradish peroxidase. J Anat.

[17] Rossi MA (2023). Control of energy homeostasis by the lateral hypothalamic area. Trends Neurosci.

[18] Rossi MA (2019). Obesity remodels activity and transcriptional state of a lateral hypothalamic brake on feeding. Science.

[19] Jennings JH (2013). The inhibitory circuit architecture of the lateral hypothalamus orchestrates feeding. Science.

[20] Jennings JH (2015). Visualizing hypothalamic network dynamics for appetitive and consummatory behaviors. Cell.

[21] Yoon NA, Diano S (2021). Hypothalamic glucose-sensing mechanisms. Diabetologia.

[22] Hausen AC (2016). Insulin-Dependent Activation of MCH neurons impairs locomotor activity and insulin sensitivity in obesity. Cell Rep.

[23] Bakker W (2022). Acute changes in systemic glycemia gate access and action of GLP-1R agonist on brain structures controlling energy homeostasis. Cell Rep.

[24] Konner AC (2007). Insulin action in AgRP-expressing neurons is required for suppression of hepatic glucose production. Cell Metab.

[25] Lin HV (2010). Divergent regulation of energy expenditure and hepatic glucose production by insulin receptor in agouti-related protein and POMC neurons. Diabetes.

[26] Qiu J (2018). Insulin and leptin excite anorexigenic pro-opiomelanocortin neurones via activation of TRPC5 channels. J Neuroendocrinol.

[27] Qiu J (2014). Insulin excites anorexigenic proopiomelanocortin neurons via activation of canonical transient receptor potential channels. Cell Metab.

[28] Roy RK (2021). Inverse neurovascular coupling contributes to positive feedback excitation of vasopressin neurons during a systemic homeostatic challenge. Cell Rep.

[29] Shimoji K (2013). White matter alteration in metabolic syndrome: diffusion tensor analysis. Diabetes Care.

[30] Thaler JP (2012). Obesity is associated with hypothalamic injury in rodents and humans. J Clin Invest.

[31] Simmons JP (2011). False-positive psychology: undisclosed flexibility in data collection and analysis allows presenting anything as significant. Psychol Sci.

[32] Poldrack RA (2017). Scanning the horizon: towards transparent and reproducible neuroimaging research. Nat Rev Neurosci.

[33] Kullmann S (2018). Dose-dependent effects of intranasal insulin on resting-state brain activity. J Clin Endocrinol Metab.

[34] Kobayashi S (2011). Comparison of relative validity of food group intakes estimated by comprehensive and brief-type self-administered diet history questionnaires against 16 d dietary records in Japanese adults. Public Health Nutr.

[35] Kobayashi S (2012). Both comprehensive and brief self-administered diet history questionnaires satisfactorily rank nutrient intakes in Japanese adults. J Epidemiol.

[36] Murase N (2002). International standardization of physical activity level: reliability and validity study of the Japanese version of the International Physical Activity Questionnaire (IPAQ). J Health Welfare Stat.

[37] Craig CL (2003). International physical activity questionnaire: 12-country reliability and validity. Med Sci Sports Exerc.

[38] Feinberg DA (2010). Multiplexed echo planar imaging for sub-second whole brain FMRI and fast diffusion imaging. PLoS One.

[39] Xu J (2013). Evaluation of slice accelerations using multiband echo planar imaging at 3 T. Neuroimage.

[40] Andersson JL (2003). How to correct susceptibility distortions in spin-echo echo-planar images: application to diffusion tensor imaging. Neuroimage.

[41] Jensen MD (2014). 2013 AHA/ACC/TOS guideline for the management of overweight and obesity in adults: a report of the American College of Cardiology/American Heart Association Task Force on Practice Guidelines and The Obesity Society. Circulation.

[42] Power JD (2012). Spurious but systematic correlations in functional connectivity MRI networks arise from subject motion. Neuroimage.

[43] Margulies DS (2007). Mapping the functional connectivity of anterior cingulate cortex. Neuroimage.

[44] Cohen AL (2008). Defining functional areas in individual human brains using resting functional connectivity MRI. Neuroimage.

[45] Buckner RL (2011). The organization of the human cerebellum estimated by intrinsic functional connectivity. J Neurophysiol.

[46] Eickhoff SB (2015). Connectivity-based parcellation: Critique and implications. Hum Brain Mapp.

[47] Laumann TO (2015). Functional system and areal organization of a highly sampled individual human brain. Neuron.

[48] Glasser MF (2016). A multi-modal parcellation of human cerebral cortex. Nature.

[49] Gordon EM (2016). Generation and evaluation of a cortical area parcellation from resting-state correlations. Cereb Cortex.

[50] Osada T (2021). Parallel cognitive processing streams in human prefrontal cortex: Parsing areal-level brain network for response inhibition. Cell Rep.

[51] Matthews DR (1985). Homeostasis model assessment: insulin resistance and beta-cell function from fasting plasma glucose and insulin concentrations in man. Diabetologia.

[52] Adams-Huet B (2014). Increased adipose tissue insulin resistance in metabolic syndrome: relationship to circulating adipokines. Metab Syndr Relat Disord.

[53] Asano S (2023). Reduced gray matter volume in the default-mode network associated with insulin resistance. Cereb Cortex.

[54] Araki E (2020). Japanese clinical practice guideline for diabetes 2019. J Diabetes Investig.

[55] Murata S (2022). Comparison of brain volume measurements made with 0.3- and 3-T MR Imaging. Magn Reson Med Sci.

