# Supplementary material for: Hypothalamic insulin resistance in type 2 diabetes is localized to the posterior hypothalamus
Source: JCI Insight. 2026 Jun 8;11(11):e198707. doi: 10.1172/jci.insight.198707 (PMC13313559; doi:10.1172/jci.insight.198707)

Supplemental Table 1. Association between LHAp at 5 and 10 minutes and metabolic parameters after adjustment for glycemic status

	LHAp (5)			LHAp (10)		
	B	95%CI	P value	B	95%CI	P value
Age (years)	2.90	-1.07—6.86	0.147	0.43	-3.09—3.95	0.807
BMI (kg/m^2)	6.39	-6.03—18.8	0.304	-1.58	-12.4—9.28	0.770
Percent body fat (%)	3.66	-0.91—8.24	0.114	0.94	-3.13—5.02	0.642
Fasting plasma glucose (mg/dL)	1.29	0.46—2.11	0.003	0.85	0.09—1.60	0.029
HbA_{1C} (%)	39.0	5.68—72.2	0.023	20.8	-9.24—50.8	0.169
Fasting serum insulin (μU/mL)	0.68	-4.80—6.17	0.802	-1.58	-6.29—3.14	0.502
Triglycerides (mg/dL)	0.10	-0.03—0.24	0.138	0.08	-0.04—0.19	0.207
HDL-C (mg/dL)	0.37	-1.15—1.89	0.627	-0.32	-1.64—0.99	0.621
Leptin (ng/ml)	2.60	-1.39—6.60	0.194	-0.25	-3.77—3.27	0.886
HOMA-IR	7.06	-8.85—23.0	0.374	-0.85	-14.7—13.0	0.902
Adipo-IR	0.00	-0.01—0.01	0.473	0.00	-0.01—0.01	0.931

B and 95% CI are expressed per 10^{-4} units.

Abbreviations: LHAp, posterior region of the lateral hypothalamic area; CI, Confidence Interval; HbA1c, glycosylated hemoglobin; HDL-C, High-density lipoprotein cholesterol; HOMA-IR, homeostasis model assessment of insulin resistance; Adipo-IR, adipose insulin resistance index.

Supplementary Table 2. Clinical characteristics of the study participants in Study 2.

	Non-DM	DM	P value
Number of subjects	1400	209	
Female (%)	60.3%	39.7%	<0.001
Age	73.0±5.4	74.2±5.3	0.002
Body Mass Index (kg/m^2)	22.6±3.0	23.9±3.3	<0.001
Percent Body Fat (%)	28.1±7.2	29.3±7.4	0.027
Skeletal Muscle Mass Index (kg/m^2)	6.4±1.0	6.7±1.0	<0.001
Subcutaneous fat area (cm2)	148.4±58.3	149.2±61.2	0.842
Visceral fat area (cm2)	75.0±36.9	101.6±42.8	<0.001
Hypertension (%)	64.1%	78.9%	<0.001
Dyslipidemia (%)	60.6%	78.0%	<0.001
Cerebrovascular disease (%)	3.9%	5.3%	0.454
Ischemic heart disease (%)	3.5%	12.0%	<0.001
MMSE	27.7±1.9	27.6±2.1	0.515
Physical Activity (METs/week)	44.4±49.1	40.7±41.9	0.296
Sedentary time (hours/day)	6.0±3.6	6.3±3.8	0.249
Energy intake (kcal)	1968.7±603.0	1923.2±558.1	0.305
Fasting plasma glucose (mg/dL, mmol/L)	96.0±9.2<br>(5.33±0.51)	130.6±23.3<br>(7.25±1.29)	<0.001
HbA_{1C} (%, mmol/mol)	5.7±0.3<br>(38.8±3.3)	6.9±0.7<br>(52.9±7.6)	<0.001

Continuous variables are presented as mean ± standard deviation, and categorical variables are shown as percentages.

P value: an unpaired *t* test or Mann-Whitney *U* test for continuous variables and chi-square tests for categorical variables, as appropriate.

Abbreviations: HbA1c, glycosylated hemoglobin; MMSE: Mini-Mental State Examination.

Supplementary Table 3. Antidiabetic treatment profile of Study 2

Medication	n	%
DPP-4 inhibitor	112	53.6%
Metformin	56	26.8%
Sulfonylurea	46	22.0%
α-glucosidase inhibitor	26	12.4%
Pioglitazone	11	5.3%
Glinide	11	5.3%
SGLT2 inhibitor	10	4.8%
Insulin	4	1.9%

Supplementary Figure 1. Time courses of BOLD signal responses in the lateral hypothalamic area following intranasal insulin administration

(A) Coronal slice displaying subregions in the LHA: anterior lateral hypothalamic area (LHAa, dark red), tuberal lateral hypothalamic area (LHAt, light blue), and posterior lateral hypothalamic area (LHAp, light green)
(B-D) Percent signal change (PSC; mean ± SEM) from 0 to 30 min relative to intranasal insulin administration (vertical dashed line at 0 min) for the control group (▲) and the type 2 diabetes (T2D) group (■) : (B) LHAa, (C) LHAt, and (D) LHAp.
* $p < 0.05$, ** $p < 0.01$ T2D versus control at the corresponding time point.

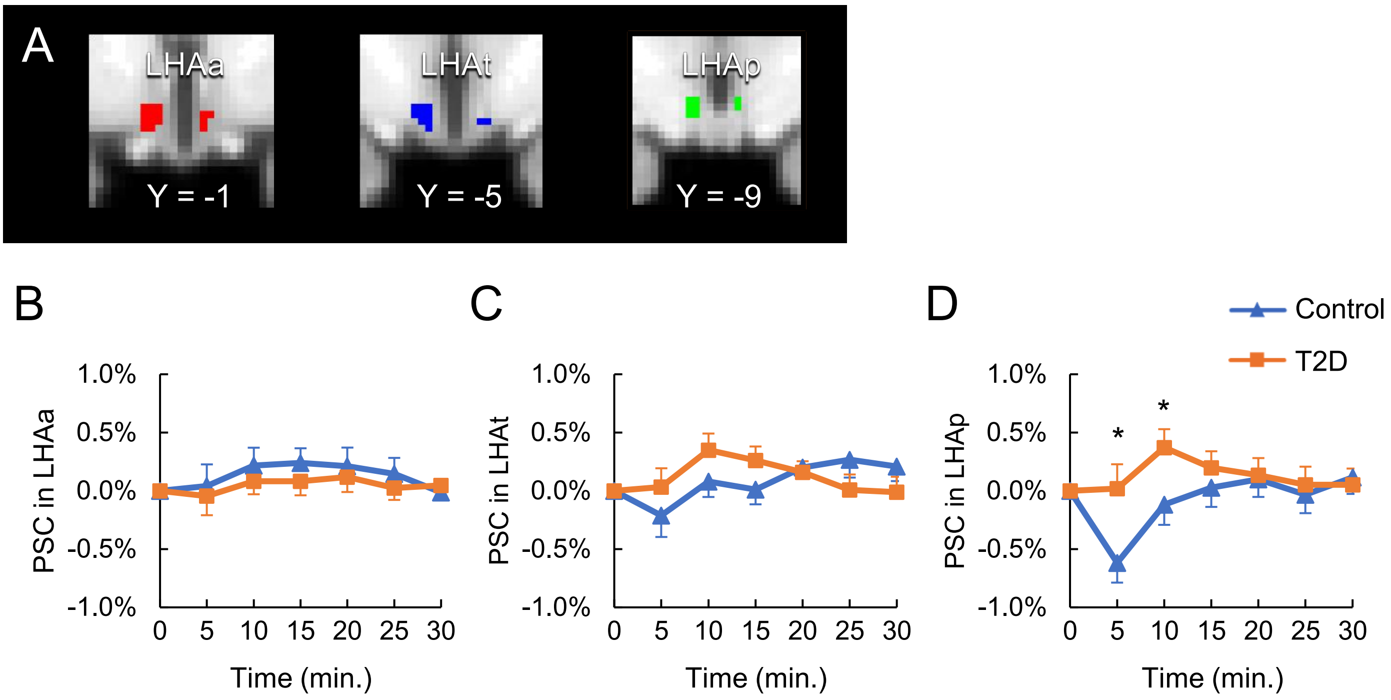

Supplementary Figure 2. Sanity-check analyses confirming the robustness of the early signal suppression in the PH.

(A) Sensitivity analysis performed in native space. The fMRI signals were extracted after inversely transforming the primary MNI-derived PH ROI into each participant's native space.
(B) Anatomically anchored analysis. The fMRI signals were extracted using an independent, macroscopic anatomical mask of the posterior hypothalamus derived from a high-resolution structural atlas (Neudorfer et al., 2020).
* $p < 0.05$, ** $p < 0.01$ T2D versus control at the corresponding time point.

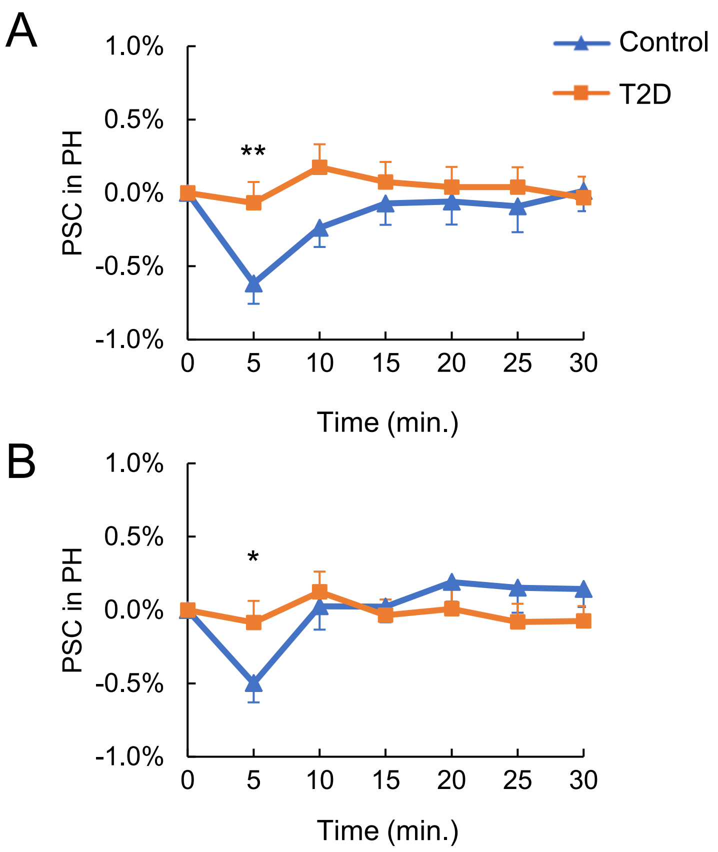

Supplementary Figure 3. Time courses of BOLD signals for intranasal distilled water administration.

Comparison of percent signal change in the PH between the insulin-treated healthy control group and an independent healthy cohort treated with intranasal distilled water.

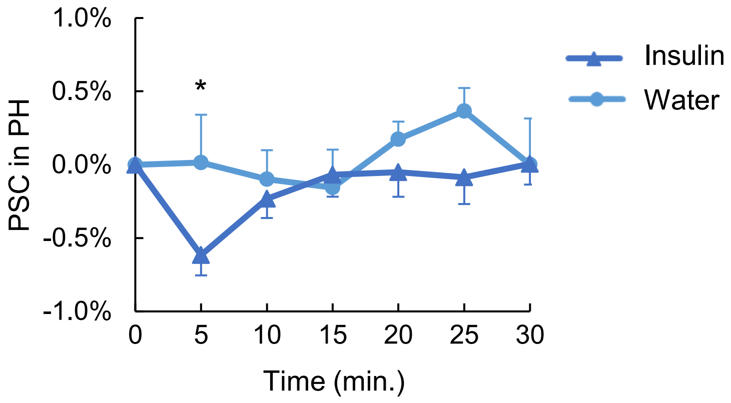

Supplementary Figure 4. Sanity-check analyses confirming the robustness of the early signal suppression in the PH.

(A) Box plots comparing gray matter volume in each subdivision (anterior, tuberal, and posterior) between the type 2 diabetes (T2D) and control groups from Study1.
(B) Box plots showing the percent signal change in hypothalamic subdivisions for the T2D and control groups from Study 1.

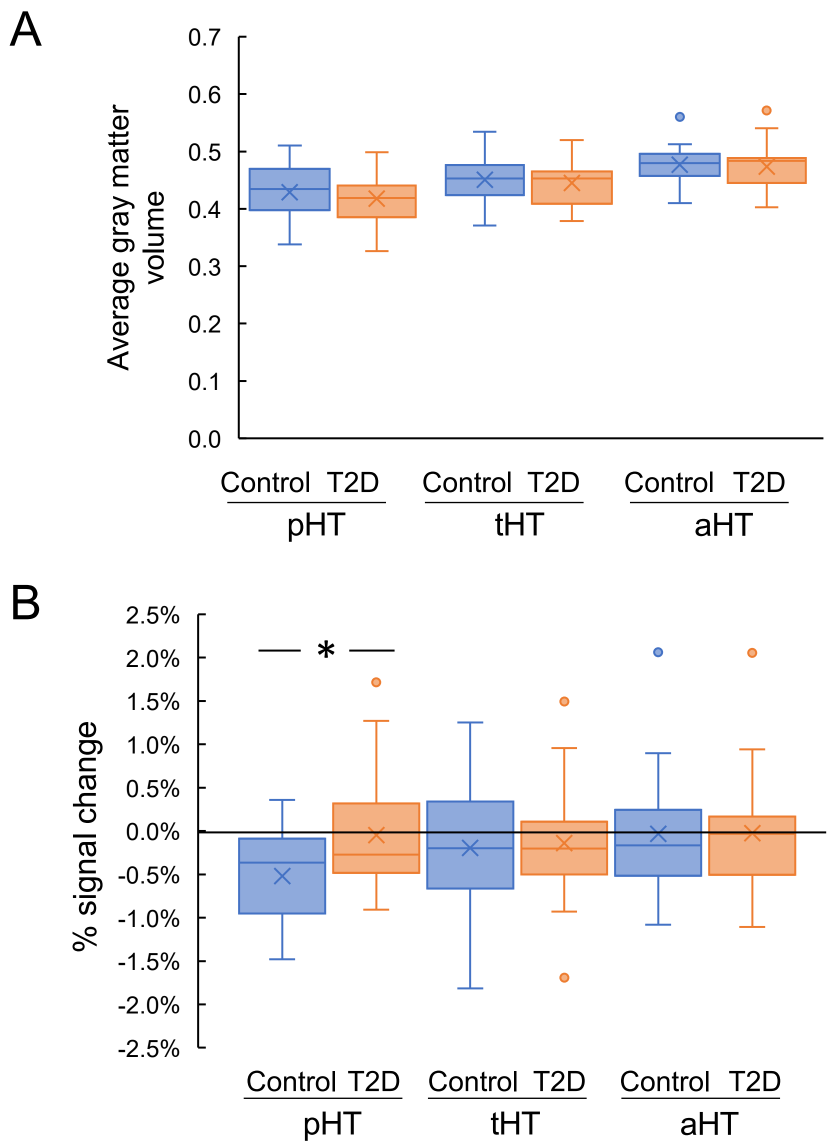

Supplement: Supplemental data [file jciinsight-11-198707-s175.pdf]
